# Long COVID among Children: Persistence of Symptoms 12 Weeks and More in a Cohort Study from Turkey: Öykü Turunç

**DOI:** 10.1093/eurpub/ckac129.252

**Published:** 2022-10-25

**Authors:** Ö Turunç, AN Emecen, AF Süner, S Keskin, E Başoğlu, N Şiyve, N Belet, H Karaoğlu Asrak, M Duman, B Ünal

**Affiliations:** 1 Department of Public Health, Dokuz Eylul University Faculty of Medicine, Izmir, Turkey; 2 Department of Pediatrics, Dokuz Eylul University Faculty of Medicine, Izmir, Turkey

## Abstract

**Background:**

COVID-19 usually cause a mild infection among children with a low fatality rate. On the other hand, increasing evidence suggests that children may have prolonged symptoms related to COVID-19. This study aims to describe the persistence of COVID-19 symptoms up to 12 or more weeks among children and to investigate associated factors including perceived socioeconomic status and parents’ education level.

**Methods:**

The study group consisted of 759 cases aged <18 years detected as SARS-CoV-2 RT-PCR positive in DEU Hospital between March 2020, and May 2021. Interviews were conducted at 1st 3rd and 6th month of diagnosis. The ongoing self-reported symptoms 12 or more weeks after infection was the dependent variable. Multivariate logistic regression models were used to evaluate associated factors with long COVID, and robust clustering using links algorithm was used to assess long COVID symptoms clusters.

**Results:**

Among 759 COVID-19 cases, 22 children were hospitalized, and 4 died. 9.6% of the children had at least one symptom related to COVID-19 after 12 weeks of the diagnosis, Symptom duration was minimum 84 days, maximum 344 days (mean±SD: 160±68 days). The most frequent symptoms were fatigue, muscle-joint pain, headache, and loss of smell and/or taste. In multivariate analysis, female gender (OR:2,3 95%CI:1.1-3.6) and symptomatic onset (OR:2,7 95%CI:1.7-20.9) were related to increased risk of long COVID. Age, long-term health conditions, socioeconomic status and mother's education level did not predict the risk of long COVID. No cluster of symptoms was found.

**Conclusions:**

About 10% of children suffer from symptoms related to COVID-19 for up to six months. Female gender and symptomatic onset of disease increased the risk of prolonged symptoms. Socioeconomic status and mother's education level was not associated with the risk of long COVID, but the evidence of the effect of social determinants of health on the outcomes of COVID-19 among children is still needed.

**Key messages:**

• One out of ten children may suffer from long COVID represents symptoms such as fatigue, headache, and muscle and joint pain. Girls and children with symptomatic onset have a higher risk of long COVID.

• The effects of social determinants on the susceptibility and outcomes of COVID-19, including death, were well studied among the adult population. There is a need for sound evidence for children.

